# Single Nasal Bones: A Report of Two Cases

**DOI:** 10.7759/cureus.71806

**Published:** 2024-10-18

**Authors:** Piyakarn Boontem, Arada Chaiyamoon, Athikhun Suwannakhan, Laphatrada Yurasakpong, Sitthisak Thonrong, Hirotomo Dochi, Joe Iwanaga, R. Shane Tubbs

**Affiliations:** 1 Division of Anatomy, University of Phayao, Phayao, THA; 2 Department of Anatomy, Faculty of Medicine, Khon Kaen University, Khon Kaen, THA; 3 Department of Anatomy, Faculty of Science, Mahidol University, Bangkok, THA; 4 Division of Otolaryngology - Head and Neck Surgery, Kanazawa University, Kanazawa, JPN; 5 Department of Neurosurgery, Tulane University School of Medicine, New Orleans, USA; 6 Department of Neurosurgery, Ochsner Neuroscience Institute, Ochsner Health System, New Orleans, USA

**Keywords:** anatomy, bony suture, cadaver, facial fractures, nasal bone

## Abstract

The nasal bones are important bony parts of the external nose and maxillofacial scaffold. Generally, the nasal bone is a small quadrangular plate paired and attached to the frontonasal suture superiorly and the nasal septum internally. The nasal septum is symmetrically located and comprises the septal cartilage, the perpendicular plate of the ethmoid bone, and the vomer. Herein, we report the anatomy of single nasal bones (SNB) identified in two human skulls. Two adult human skulls were found to have SNB. In one, the nasal septum was bifid and hooked as it joined with the nasal bone superiorly. Understanding the morphological variations of the nasal bone and septum is important for maxillofacial surgeries and should be further clarified and studied. Although apparently very rare, a SNB, as seen in the present two cases, should be considered when interpreting imaging of the face.

## Introduction

The nasal bone is an essential bony component of the external nose and maxillofacial scaffold. The nasal bones are small, paired, symmetrical quadrangular plates. Each nasal bone is articulated in the midface to form the internasal suture. The point where the internasal septum meets the frontal bone is called the nasion and is an important point in determining cephalometric landmarks [1]. Superiorly, both nasal bones articulate with the frontal bone to form the frontonasal sutures. Laterally, the nasal bones are attached to the frontal process of the maxillae to form nasomaxillary sutures. The internal surface of the nasal bone is concave and attached to the nasal septum. The nasal septum is made up of the quadrangular cartilage anteriorly, the perpendicular plate of the ethmoid superiorly, the vomer posteroinferiorly, and the crest of the maxillary and palatine bones inferiorly [2].

The nasal bone is perforated by the nasal foramen (foramen nasale), which serves as passage vessels and nerve fibers. Furthermore, the external surface of each nasal bone is covered by the procerus and nasalis muscles [3].

Regarding the development of the pharyngeal arch, including nasal bone formation, the nasal bone undergoes two stages of differentiation. It is formed in the membrane of the dense mesenchyme overlying the cartilaginous nasal capsule. The first visible primitive nasal bone is at 9-10 weeks [4,5]. Thus, the authors aim to study and clarify the morphologies and morphometrics of the single nasal bone (SNB) found in a Thai and Caucasian skull.

## Case presentation

During routine examinations of the skulls, two skulls with an SNB were found. The first (Case 1) was found in the skull collection at the Division of Anatomy, School of Medical Science, University of Phayao, Thailand (ethical approval number: HREC-UP-HSST 1.1/001/68), and the second (Case 2) was identified in the Haman-Todd Human Osteological Collection in the Cleveland Museum of Natural History, Cleveland, Ohio, USA. Measurements of the SNBs were made using a digital microcaliper (Mitutoyo, Kanagawa, Japan).

**Figure 1:**
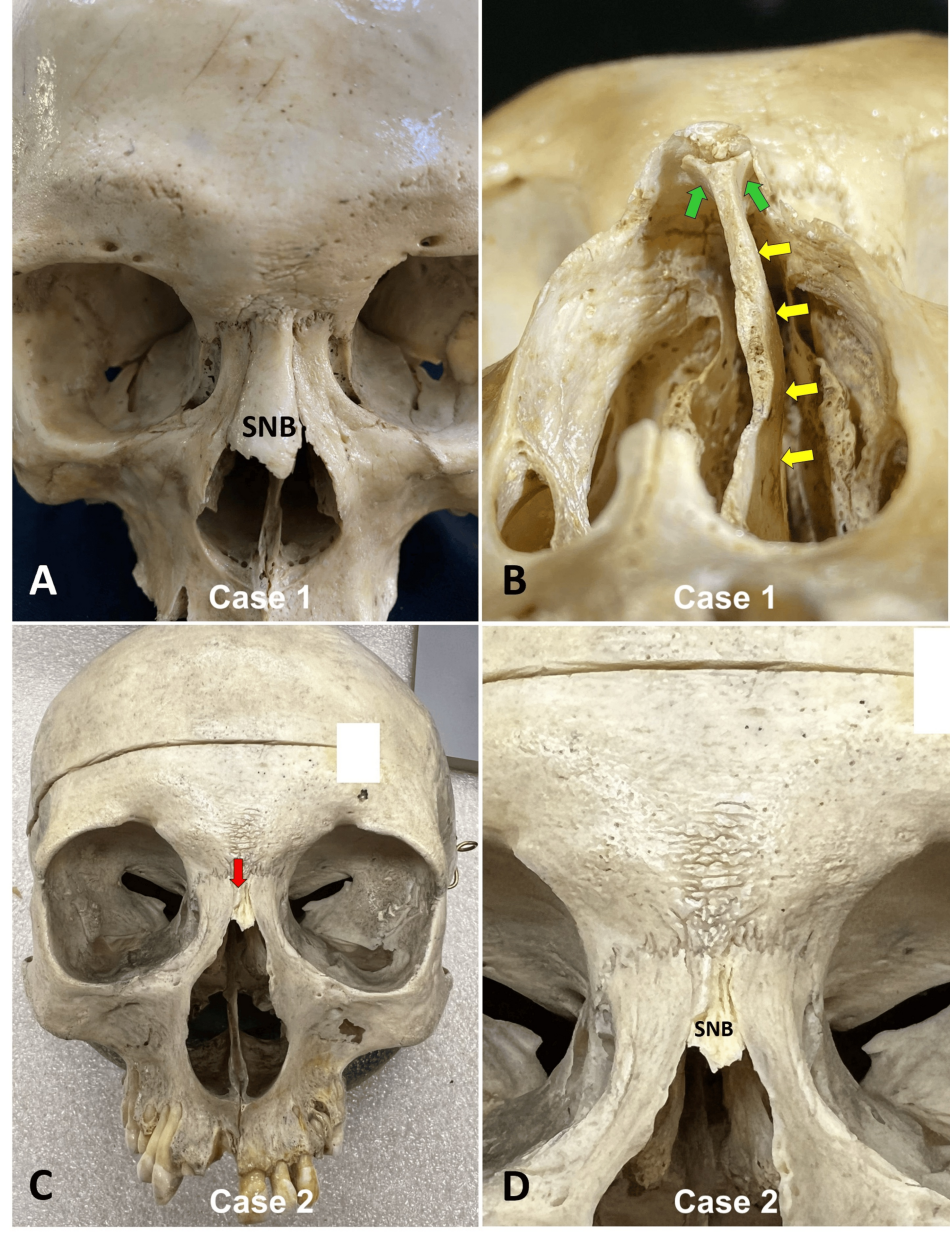
Single nasal bones (SNB) in Thai (A and B) and Caucasian (C and D) skulls A: Anterior view of the Thai skull (Case 1). Note that the SNB was located between the frontal processes of the maxillae, but the internasal suture is absent. B: Inferior view of the Thai skull (Case 1). Note that the anterior edge of the septum (yellow arrow) is bifid (green arrow), and the SNB has a hook shape articulated with the septum's bifid part. C and D: Anterior view of the Caucasian skull (Case 2). The red arrow shows no internasal suture.

For Case 1, the skull was a male specimen (unknown age). The width of the SNB (Figure 1), upper and lower parts, was 6.82 mm and 12.08 mm, respectively.

The maximum length of the SNB was 23.03 mm. Notably, the left nasal cavity was smaller than the right side due to the deviation of the nasal septum. The diameters of the left and right nasal cavities were 9.97 mm and 14.31 mm, respectively. The anterior portion of the nasal septum was bifid and hooked with the nasal bone. A nasal foramen was absent in this skull. There were no other abnormal fusions of the sutures of this skull.

For Case 2, the skull was from a 60-year-old at-death Caucasian male specimen. The SNB was 4.15 mm wide at its midpart and had a length of 8.50 mm (Figure 1). Its distal part was irregular in shape with a central pointed part. No nasal foramen was located in this SNB. The bony septum was fractured, and no other major anatomical variations were noted in this specimen.

The authors state that every effort was made to follow all local and international ethical guidelines and laws that pertain to the use of human cadaveric donors in anatomical research [6].

## Discussion

Understanding nasal bone anatomy is very important for planning rhinoplastic procedures. During the embryonic period, the nasal bone develops in the membrane in the dense mesenchyme overlying the cartilaginous nasal capsule. They are first visible at 9-10 weeks [4,5]. In normal euploid fetuses, nasal bone length increases with advancing gestational age. An absent nasal bone in any trimester is a potential marker for fetal aneuploidy [7]. The human internasal suture, missing in the present cases, begins its closure in the 20s and completely closes in the 30s [8,9]. In one of the current cases, the nasal bone in the single plate deviated to the right side of the nose. A SNB may develop due to malformations comprising suture and cartilage development [10]. Both sides of the nasal bones might fuse into an SNB. One nasal bone that disappeared from the prenatal screening aneuploidy in the first and second trimesters was at high risk of trisomies [11]. The absence of the internasal suture presented in these cases might be due to the fusion of the right and left nasal bones (acquired) or congenital. As with other sutures in the body, aging might be one of the reasons for the fusion. However, there is no such evidence for an internasal suture.

Deviated septum with a SNB

A previous study reported that nose malformation can significantly affect a child's well-being [12]. Upper respiratory obstruction may result from nasal septum deviation [13]. Hence, the one case reported herein with a deviated septum might have had upper airway problems. The cause of the deviation is unknown, but the external force on the SNB without an internasal suture might compress the septum that might have developed the deviation.

Nasal bone fracture (NBF)

The nasal bones are the most commonly fractured facial bones, accounting for up to 45% of facial fractures, followed by the mandible [14]. NBF is often found with other facial fractures, such as maxilla, frontal bone, and nasal septum, which might complicate diagnosis and treatment. Fractures of the internasal suture are one of the most common locations of the NBF because of the distribution of the stress. If the patients with SNB have an NBF, the stress might not be distributed, leading to other complicated facial fractures due to a lack of the internasal suture.

## Conclusions

The nasal bone is a crucial component of the midface region. Although there is only a little evidence of this variation, understanding the variations of the nasal bones should be further clarified and studied. Sutures can be very small and thin, making it difficult to accurately assess their presence or absence in imaging modalities such as computed tomography. A larger collection of dry skulls indeed offers an advantage for the research.

## References

[1] Hegazy AA (2021). Skull sutures as anatomical landmarks. The sutures of the skull: anatomy, embryology, imaging, and surgery.

[2] Kim L, Huddle MG, Smith RM, Byrne P (2020). Nasal fractures. Facial trauma Surgery.

[3] Hefner JT, Linde KC (2018). Nasal bone contour. Atlas of human cranial macromorphoscopic traits.

[4] Sandikcioglu M, Mølsted K, Kjaer I (1994). The prenatal development of the human nasal and vomeral bones. J Craniofac Genet Dev Biol.

[5] Macklin CC (1914). The skull of a human fetus of 40 mm. Am J Anat.

[6] Iwanaga J, Singh V, Takeda S (2022). Standardized statement for the ethical use of human cadaveric tissues in anatomy research papers: recommendations from anatomical journal editors-in-chief. Clin Anat.

[7] Benacerraf BR, Bromley B, Jelin AC (2019). Absent nasal bone. Am J Obstet Gynecol.

[8] Wang MM, Haveles CS, Zukotynski BK, Reid RR, Lee JC (2022). The 27 facial sutures: timing and clinical consequences of closure. Plast Reconstr Surg.

[9] 8] Sivakumaran R (2024). Critical Analysis of the Factors Affecting the “Cranial Suture Aging Method” Using the Hamann Todd Collection. using the Hamann Todd Collection.

[10] Kjaer I, Keeling JW, Fischer Hansen B, Becktor KB (2002). Midline skeletodental morphology in holoprosencephaly. Cleft Palate Craniofac J.

[11] Kashyap V, Kashyap N, Khanna S, Verma S, Kashyap A (2021). Absence of one nasal bone is better detected in transverse scans as a missing limb of inverted “V” sign. Ultrasound Obstet Gynecol.

[12] Neskey D, Eloy JA, Casiano RR (2009). Nasal, septal, and turbinate anatomy and embryology. Otolaryngol Clin North Am.

[13] Teul I, Slawinski G, Lewandowski J, Dzieciolowska-Baran E, Gawlikowska-Sroka A, Czerwinski F (2010). Nasal septum morphology in human fetuses in computed tomography images. Eur J Med Res.

[14] Mondin V, Rinaldo A, Ferlito A (2005). Management of nasal bone fractures. Am J Otolaryngol.

